# Prognostic factors in young patients with oral cavity cancer: a systematic review and meta-analysis of 24 studies

**DOI:** 10.3389/fonc.2026.1780074

**Published:** 2026-02-25

**Authors:** Rami Saade, Rita Khoury, Jana Hassan, Gibran Atwi, Hady Ghanem, Caroline Jabbour, Annoir Shayya

**Affiliations:** 1 Lebanese American University Gilbert and Rose-Marie Chagoury, School of Medicine, Byblos, Lebanon; 2 Department of Otolaryngology Head and Neck Surgery, Lebanese American University Medical Center, Beirut, Lebanon; 3 Department of Internal Medicine, Division of Hematology Oncology, Lebanese American University Medical Center, Beirut, Lebanon; 4 Radiation Oncology, Lebanese American University Medical Center, Beirut, Lebanon

**Keywords:** hazard ratio, meta-analysis, prognostic factors, survival, systematic review, tongue cancer, young-onset oral cancer

## Abstract

**Background:**

Young-onset oral cancer is increasingly recognized as a distinct clinical entity, yet prognostic determinants in this population remain poorly defined. This systematic review and meta-analysis aimed to identify and synthesize prognostic factors associated with overall survival among young patients with oral and tongue cancers.

**Methods:**

A comprehensive search of PubMed, Scopus, and Web of Science was conducted on September 23, 2024. Eligible studies included observational cohorts reporting regression-derived prognostic estimates in young patients with oral cancer. Adjusted hazard ratios (aHRs) were pooled using random-effects models with restricted maximum likelihood, whereas unadjusted estimates were narratively summarized. Risk of bias was assessed using the NIH Quality Assessment Tool. Subgroup analyses were not feasible due to limited stratified reporting, and publication bias was not evaluated because all pooled analyses contained fewer than ten studies.

**Results:**

Twenty-four studies encompassing 6,965 young patients were included. Several demographic factors showed no significant association with survival, including age and sex, while Black race was associated with worse outcomes (aHR = 2.79, 95% CI 1.40–5.56). Tumor characteristics linked to poorer prognosis included larger tumor size (aHR = 1.01 per cm, 95% CI 1.00–1.03) and greater depth of invasion (aHR = 1.03, 95% CI 1.01–1.05). High-grade tumors (grade 3–4) (aHR = 2.15, 95% CI 1.52–2.77) and poorly differentiated histology (aHR = 7.75, 95% CI 2.61–23.01) demonstrated strong adverse prognostic associations. Nodal disease significantly increased risk, including higher N stage (aHR = 1.24, 95% CI 1.11–1.37) and N+ status (aHR = 2.05, 95% CI 1.24–2.85). Single-study findings—such as TERTp mutation (aHR = 3.00), PRKCA mutation (aHR = 3.57), Stage IVB, disease recurrence, and several treatment-related variables—suggest possible associations but remain inconclusive.

**Conclusions:**

Among young patients with oral and tongue cancer, nodal involvement, high-grade or poorly differentiated tumors, increased depth of invasion, and larger tumor size were the most consistently associated with poorer survival. Evidence for molecular and treatment-related factors is limited and requires further validation. These findings highlight the need for standardized reporting and prospective studies tailored to young-onset disease.

## 1 Introduction

Oral squamous cell carcinoma (OSCC) remains a significant global health burden, accounting for more than 350,000 new cases and 177,000 deaths annually (1). Although OSCC traditionally affects individuals in their sixth and seventh decades of life, an increasing proportion of cases now occur in younger adults, often defined as ≤40 or ≤45 years (2, 3). This epidemiologic shift has been documented across multiple geographic regions and has raised substantial clinical interest because young patients frequently present without classical risk factors, especially tobacco and alcohol exposure (4, 5). The biological underpinnings of OSCC in younger adults remain incompletely understood, prompting growing concern that this subgroup may represent a clinically and molecularly distinct disease entity.

Importantly, accumulating evidence suggests that OSCC arising in younger patients may be associated with unfavorable clinical behavior and poorer oncologic outcomes compared with disease in older adults. Several retrospective cohorts have reported higher local recurrence rates, increased locoregional failure, and inferior disease-specific survival in younger patients despite comparable or even earlier-stage presentation (6–11). These observations challenge the historical assumption that younger age confers a prognostic advantage and raise concern that early-onset OSCC may represent a more aggressive disease phenotype rather than simply an age-shifted version of conventional OSCC.

Nonetheless, the prognostic implications of young age remain controversial. While some studies suggest survival outcomes in younger patients are similar to or better than those in older cohorts (12, 13), others consistently demonstrate worse outcomes, particularly with respect to recurrence and cancer-specific mortality (14, 15). Systematic reviews and meta-analyses mirror this inconsistency: some conclude that younger age is not an independent adverse factor (2), whereas others identify younger patients as having higher local recurrence risk despite similar overall survival (3). Collectively, these inconsistencies underscore that young-onset OSCC cannot be assumed to behave indolently and, in fact, may carry a prognostic disadvantage that remains insufficiently characterized.

A particularly important challenge in the literature is the limited availability of adjusted prognostic estimates. Many prior analyses rely on crude survival comparisons that do not account for confounding by tumor stage, nodal burden, margin status, or treatment modality—factors strongly associated with outcome in OSCC regardless of age (16). Moreover, reports focusing specifically on young-onset OSCC often pool diverse oral subsites, despite well-established evidence that tongue cancer, especially in younger populations, may behave differently from other oral cavity subsites (14). Another major gap is the limited and inconsistent investigation of molecular characteristics such as TERT promoter mutations or PRKCA alterations, which have been implicated in the biology of early-onset OSCC but remain underexplored and rarely evaluated in multivariable frameworks (14, 15).

These uncertainties underscore the importance of establishing a robust, evidence-based understanding of prognostic factors in young-onset OSCC, grounded in adjusted regression models rather than univariate descriptions. Given that younger patients often experience significant long-term functional morbidity from surgical and adjuvant treatments, accurate prognostic assessment is essential for risk-adapted management. Determining whether established clinicopathologic and molecular predictors carry similar weight in younger patients—and whether young age itself influences survival—has direct implications for treatment decision-making, survivorship expectations, and future development of age-specific prognostic tools.

Therefore, this systematic review and meta-analysis aimed to synthesize the highest-quality regression-derived prognostic evidence for young-onset oral cancer, focusing strictly on studies reporting adjusted hazard ratios. By dissecting demographic, clinicopathologic, molecular, and treatment-related predictors, this study seeks to clarify the prognostic landscape of young-onset OSCC and address the persistent gaps in the literature regarding whether this rising patient subgroup represents a biologically distinct entity with unique prognostic determinants.

## 2 Methods

### 2.1 Study design and reporting framework

This systematic review and meta-analysis was conducted in accordance with the Preferred Reporting Items for Systematic Reviews and Meta-Analyses (PRISMA) 2020 guidelines (17). The protocol was developed *a priori* and followed established recommendations for prognostic factor research synthesis.

### 2.2 Search strategy

A comprehensive literature search was performed on September 23, 2024, across PubMed, Scopus, and Web of Science. The search strategy combined controlled vocabulary and free-text keywords related to young age, oral and tongue cancers, and prognostic or regression-based outcomes. The exact search queries used for each database are provided in **Supplementary Table S1**. No restrictions were applied on publication year. Only English-language publications were considered due to feasibility constraints. All retrieved records were imported into EndNote for reference management, and duplicates were removed prior to screening.

### 2.3 Eligibility criteria

Studies were eligible for inclusion if they met the following criteria:

- involved patients with young-onset squamous cell carcinoma of the oral cavity, defined according to each study’s prespecified age cutoff;
- reported prognostic associations (with overall survival) using regression-based measures (e.g., adjusted or unadjusted hazard ratios);
- included at least 20 patients to ensure minimum analytic robustness; and
- used an observational cohort or registry-based design.

Studies were excluded if they:

- focused on salivary gland cancers or non-squamous oral malignancies
- did not provide prognostic analyses specific to the young patient subgroup;
- lacked prognostic factor reporting;
- included fewer than 20 participants;
- did not report regression-derived effect estimates (e.g., hazard ratios, risk ratios, or odds ratios); or
- constituted reviews, editorials, letters, or case reports.

### 2.4 Study selection

**Figure 1:**
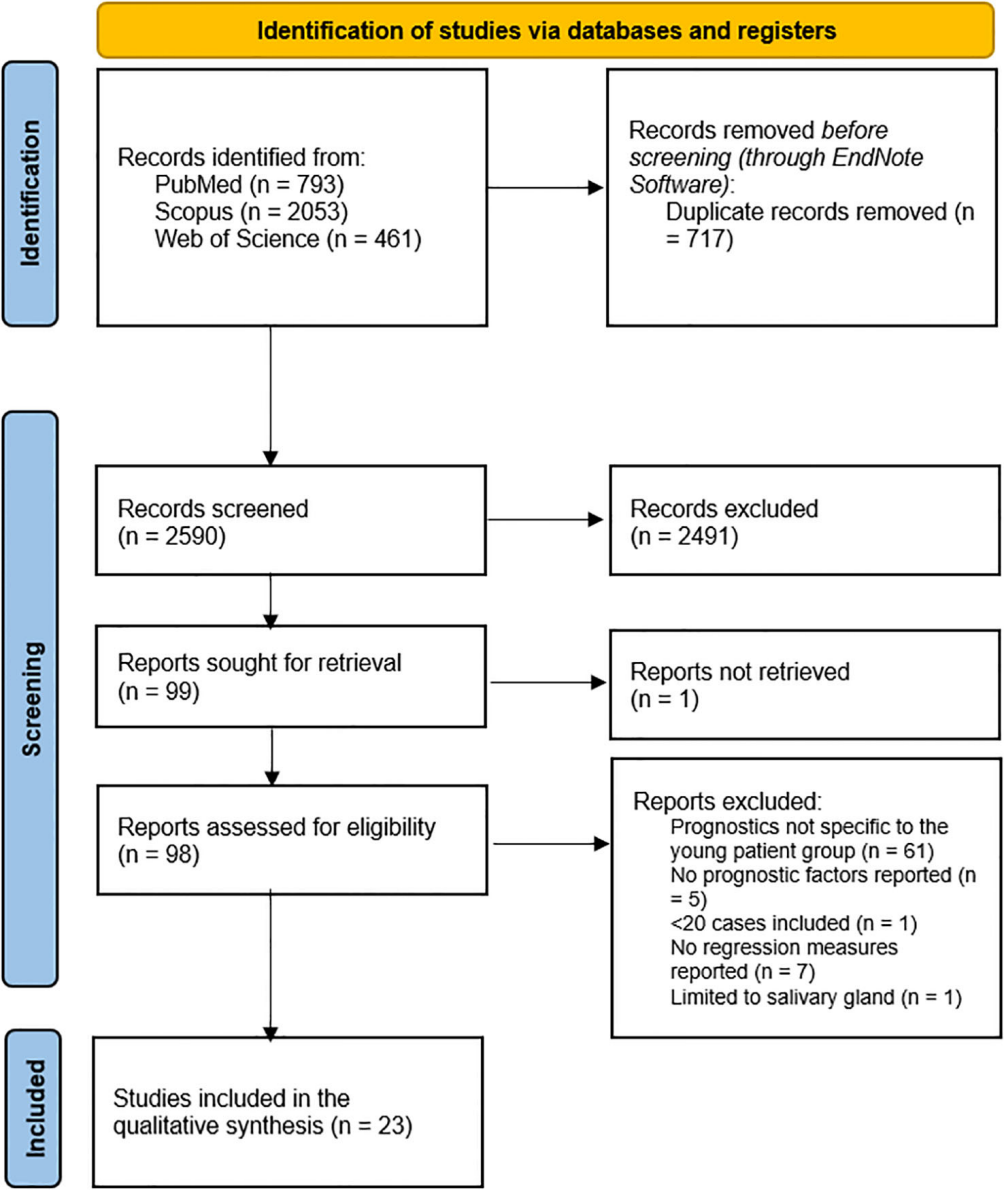
A PRISMA flow diagram showing the results of the database search.

Two reviewers independently screened titles and abstracts and subsequently reviewed the full texts of potentially eligible articles. Disagreements were resolved through discussion until consensus was achieved. The study selection process is summarized using a PRISMA flow diagram (**Figure 1**).

### 2.5 Data extraction

Data were extracted independently and in duplicate using a standardized form. Extracted variables included study characteristics (country, year, design), sample size, demographic and clinical characteristics, tumor site and stage, treatment modality, and all reported prognostic estimates. Overall survival (OS) was predefined as the primary outcome for quantitative synthesis. Adjusted estimates for disease-specific survival (DSS), disease-free survival (DFS), and other oncologic outcomes were extracted when available but were summarized narratively due to limited and heterogeneous reporting. For each prognostic factor, adjusted hazard ratios (aHRs) were extracted preferentially; unadjusted estimates were collected when adjusted values were unavailable and are presented narratively in **Supplementary Table S2**. When studies presented multiple adjusted models, the model with the most complete adjustment for confounders was selected.

### 2.6 Risk of bias assessment

Methodological quality of included studies was evaluated using the National Institutes of Health (NIH) Quality Assessment Tool for Observational Cohort and Cross-Sectional Studies. Each study was rated as good, fair, or poor quality based on criteria such as adequacy of confounding control, outcome assessment, loss to follow-up, and analytical transparency.

### 2.7 Data synthesis and statistical analysis

Quantitative synthesis focused on adjusted hazard ratios for overall survival (OS). Meta-analyses were performed using a random-effects model with restricted maximum likelihood (REML) estimation to account for between-study heterogeneity. For each prognostic factor reported in at least two studies, log-transformed hazard ratios and their standard errors were pooled and back-transformed for interpretation. Heterogeneity was quantified using the I² statistic. Adjusted prognostic estimates reported for DSS, DFS, or locoregional outcomes were not pooled and are summarized narratively in **Supplementary Table S3**.

Unadjusted effect estimates, or those reported only once in the literature, were not pooled and instead summarized narratively. Subgroup analyses based on country, geographic region, tumor location, or clinical stage could not be performed due to insufficient stratified reporting across included studies. Publication bias was not assessed because no meta-analysis contained ten or more studies, consistent with recommendations that funnel plot asymmetry tests are unreliable under this threshold.

All statistical analyses were conducted using STATA V18.

## 3 Results

### 3.1 Literature search results

The database search yielded a total of 3,307 records, including 793 from PubMed, 2,053 from Scopus, and 461 from Web of Science (**Figure 1**). After removal of 717 duplicate entries through EndNote, 2,590 unique records proceeded to title and abstract screening. Of these, 2491 were excluded, leaving 99 reports for full-text retrieval. One report could not be retrieved, resulting in 98 articles being assessed for eligibility. Following full-text evaluation, 74 reports were excluded for the following reasons: prognostic analyses not specific to the young patient group (n = 61), absence of prognostic factors (n = 5), inclusion of fewer than 20 cases (n = 1), limited to salivary gland cancer (n = 1), or lack of regression-based effect estimates (n = 7). A total of 23 studies met the inclusion criteria and were incorporated into the qualitative/quantitative synthesis (18–40).

### 3.2 Baseline characteristics of included studies

The summary of included studies’ characteristics can be found in **Table 1**. Most evidence came from the United States (9 studies, 37.50%) followed by India (5 studies, 20.83%), and South Korea (2 studies, 8.33%), respectively. All of included studies were retrospective cohort in design. Overall, 6965 young oral cancer patients were included, with male predominance (1925 patients, 56.97%) in studies reporting gender data. Among studies reporting cancer stage-related data, patients with stage I were the most predominant (931 patients, 33.1%), followed by stage II (786 patients, 27.9%), stage III (612 patients, 21.7%), and stage IV (486 patients, 17.3%), respectively. As for location-based cancer distribution, tongue cancer was the most frequently reported site (**Table 2**).

**Table 1:** Baseline characteristics of studies investigating determinants of survival in patients with young oral cavity cancer.

Author (YOP)	Country	YOI	Design	Sample	Age	Gender		Stage			
						Male	Female	I	II	III	IV
Adduri (2014) (18)	India	2008	Retrospective Study	121	≤ 45 yrs	81	40	–	–	–	–
Bommakanti (2023) (19)	USA	2005 - 2014	Retrospective Study	3262	< 45 yrs	–	–	–	–	–	–
Decker (2021) (20)	Brazil	2011 - 2016	Retrospective Study	76	≤ 60 yrs	50	26	16	16	60	60
Deneuve (2022) (21)	France	Jan 2005 - Dec 2015	Retrospective Study	185	≤ 40 yrs	105	80	70	75	21	19
Fan (2014) (22)	China	Jan 2001 - Dec 2010	Retrospective Study	100	< 45 yrs	66	34	21	28	26	25
Farhat (2022) (23)	USA	Jan 1992 - Dec 2017	Retrospective Study	80	≤ 45 yrs	44	36	32	15	16	17
Gamez (2018) (24)	USA	1980 - 2014	Retrospective Study	124	≤ 40 yrs	74	50	65	29	9	12
Iype (2001) (26)	India	1982 - 1996	Retrospective Study	264	≤ 35 yrs	184	80	–	–	–	–
Iype (2004) (25)	India	1982 - 1996	Retrospective Study	46	< 35 yrs	40	6	16	16	30	30
Kies (2012) (27)	USA	Sep 2001 - Oct 2004	Retrospective Study	23	18–49 yrs	10	13	–	14	14	9
Kim (2023) (28)	South Korea	–	Retrospective Study	44	≤ 45 yrs	29	15	–	–	–	–
Lee (2020) (29)	South Korea	–	Retrospective Study	49	≤ 45 yrs	30	19	13	5	14	17
Liao (2018) (30)	Taiwan	2007 - 2014	Retrospective Study	457	≤ 65 yrs	427	30	270	270	187	187
Manuel (2003) (31)	India	1990 - 1994	Retrospective Study	76	≤ 45 yrs	48	28	17	17	27	15
Mascitti (2020) (32)	Italy	1991 - 2018	Retrospective Study	66	< 40 yrs	51	15	–	–	–	–
Miller (2019) (33)	USA	2000 - 2016	Retrospective Study	23	22–40 yrs	17	6	12	0	9	2
Mneimneh (2021) (34)	USA	–	Retrospective Study	150	≤ 45 yrs	89	61	78	46	13	9
Myers (2000) (35)	USA	1973 - 1995	Retrospective Study	64	≤ 40 yrs	37	27	–	–	–	–
Okuyama (2021) (36)	Japan	April 2008 - March 2017	Retrospective Study	101	AYA	57	50	61	32	8	6
Parzefall (2021) (37)	Austria	–	Retrospective Study	29	≤ 45 yrs	17	12	–	–	–	–
Subramaniam (2018) (38)	India	2004 - 2014	Retrospective Study	82	≤ 45 yrs	55	27	17	20	31	14
Thomas (2012) (39)	USA	1980 - 2004	Retrospective Study	62	18–40 yrs	37	25	–	–	–	–
Warnakulasuriya (2007) (40)	UK	1986 - 2002	Retrospective Study	483	< 45 yrs	–	–	–	–	–	–

**Table 2:** Tumor location distributions of oral cavity cancer among included studies.

Author (YOP)	Tongue	FOM	Buccal mucosa	Gingiva	Hard Palate	Lip	Retromolar trigone	Alveolus	Palate	Other
Adduri (2014) (18)	–	–	–	–	–	–	–	–	–	–
Bommakanti (2023) (19)	–	–	–	–	–	–	–	–	–	–
Decker (2021) (20)	26%	35%	–	–	–	–	–	–	–	15%
Deneuve (2022) (21)	–	–	–	–	–	–	–	–	–	–
Fan (2014) (22)	63%	11%	7%	16%	3%	–	–	–	–	–
Farhat (2022) (23)	76.25%	–	3.75%	10%	–	–	3.75%	–	–	–
Gamez (2018) (24)	86.30%	–	–	–	–	–	–	–	–	13.70%
Iype (2001) (26)	52.00%	1.90%	26%	–	–	2.30%	–	10%	4.50%	3.80%
Iype (2004) (25)	36%	–	37%	–	–	–	–	–	–	–
Kies (2012) (27)	–	–	–	–	–	–	–	–	–	–
Kim (2023) (28)	–	–	–	–	–	–	–	–	–	–
Lee (2020) (29)	84%	–	–	–	–	–	–	–	–	16%
Liao (2018) (30)	36.76%	3.28%	43.33%	10.07%	2.41%	4.16%	–	–	–	–
Manuel (2003) (31)	–	–	–	–	–	–	–	–	–	–
Mascitti (2020) (32)	78.80%	4.50%	6.10%	9.10%	–	–	1.50%	–	–	–
Miller (2019) (33)	–	–	–	–	–	–	–	–	–	–
Mneimneh (2021) (34)	87%	–	5.33%	6%	–	1.33%	–	–	–	–
Myers (2000) (35)	–	–	–	–	–	–	–	–	–	–
Okuyama (2021) (36)	94.40%	3.74%	0.93%	–	0%	–	–	–	–	0%
Parzefall (2021) (37)	–	–	–	–	–	–	–	–	–	–
Subramaniam (2018) (38)	80.49%	–	19.51%	–	–	–	–	–	–	–
Thomas (2012) (39)	–	–	–	–	–	–	–	–	–	–
Warnakulasuriya (2007) (40)	–	–	–	–	–	–	–	–	–	–

FOM, floor of mouth; YOP, year of publication.

### 3.3 Methodological quality

A detailed description of each study’s methodological quality is provided in **Table 3**. Overall, nine studies had good quality while the remaining 15 studies had fair quality. The main drawback was the lack of confounding control either in the design or analysis phase.

**Table 3:** Methodological quality of included studies using the Newcastle Ottawa Scale for cohort studies.

Author (YOP)	Selection				Comparability	Outcome			Overall Quality
	Representativeness of the exposed cohort	Selection of non-exposed cohort	Ascertainment of exposure	Demonstration that outcome of interest was not present at start of study	Based on design and/or analysis	Assessment of outcome	Was follow-up long enough?	Adequacy of follow-up of cohorts	
Adduri (2014) (18)	*	*	*	*	*	*	*	*	Fair
Bommakanti (2023) (19)	*	*	*	*	**	*	*	*	Good
Decker (2021) (20)	*	*	*	*	**	*	*	*	Good
Deneuve (2022) (21)	*	*	*	*	*	*	*	*	Fair
Fan (2014) (22)	*	*	*	*	*	*	*	*	Fair
Farhat (2022) (23)	*	*	*	*	*	*	*	*	Fair
Gamez (2018) (24)	*	*	*	*	**	*	*	*	Good
Iype (2001) (26)	*	*	*	*	*	*	*	*	Fair
Iype (2004) (25)	*	*	*	*	*	*	*	*	Fair
Kies (2012) (27)	*	*	*	*	*	*	*	*	Fair
Kim (2023) (28)	*	*	*	*	**	*	*	*	Good
Lee (2020) (29)	*	*	*	*	**	*	*	*	Good
Liao (2018) (30)	*	*	*	*	*	*	*	*	Fair
Manuel (2003) (31)	*	*	*	*	*	*	*	*	Fair
Mascitti (2020) (32)	*	*	*	*	**	*	*	*	Good
Miller (2019) (33)	*	*	*	*	*	*	*	*	Fair
Mneimneh (2021) (34)	*	*	*	*	**	*	*	*	Good
Myers (2000) (35)	*	*	*	*	*	*	*	*	Fair
Okuyama (2021) (36)	*	*	*	*	*	*	*	*	Fair
Parzefall (2021) (37)	–	*	*	*	**	*	*	*	Fair
Subramaniam (2018) (38)	*	*	*	*	*	*	*	*	Fair
Thomas (2012) (39)	*	*	*	*	*	*	*	*	Fair
Warnakulasuriya (2007) (40)	*	*	*	*	**	*	*	*	Good

Quality ratings were as follows: **Good quality:** 3 or 4 stars in selection domain AND 1 or 2 stars in comparability domain AND 2 or 3 stars in outcome/exposure domain; **Fair quality:** 2 stars in selection domain AND 1 or 2 stars in comparability domain AND 2 or 3 stars in outcome/exposure domain; **Poor quality:** 0 or 1 star in selection domain OR 0 stars in comparability domain OR 0 or 1 stars in outcome/exposure domain. Each “*” equals to 1 point in the methodological quality assessment.

### 3.4 Demographic factors

**Figure 2:**
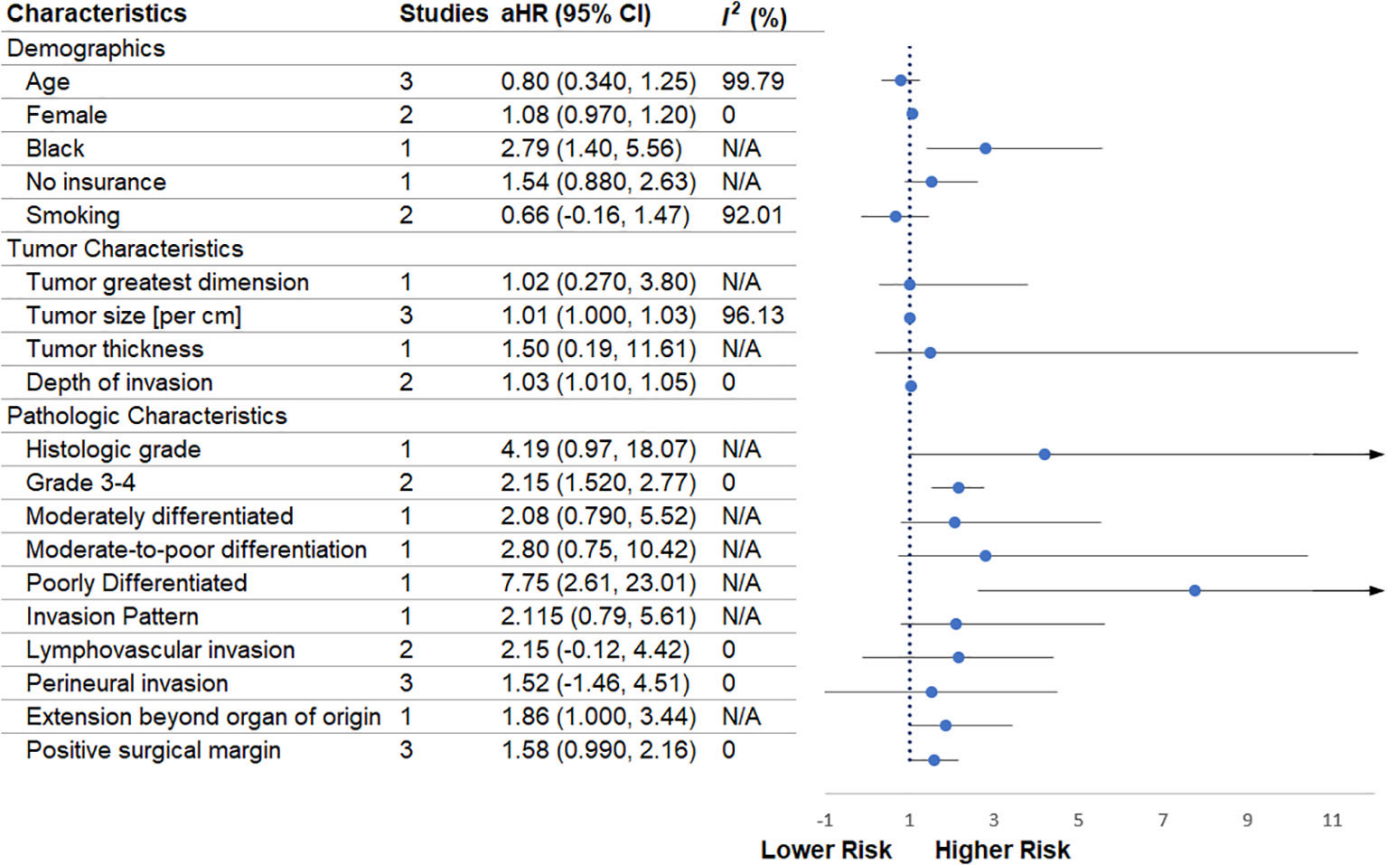
Forest plot showing the meta-analytic estimates of demographic and tumor-related prognosticators of overall survival.

Across demographic variables (**Figure 2**), age was not significantly associated with survival (three studies; aHR = 0.80, 95% CI 0.34–1.25; I² = 99.79%). Female sex similarly showed no significant effect (two studies; aHR = 1.08, 95% CI 0.97–1.20; I² = 0%). Black race demonstrated a significantly higher risk (one study; aHR = 2.79, 95% CI 1.40–5.56). Lack of insurance was evaluated in one study only (aHR = 1.54, 95% CI 0.88–2.63), and thus this observation remains inconclusive. Smoking was not significantly associated with prognosis (two studies; aHR = 0.66, 95% CI –0.16 to 1.47; I² = 92.01%).

### 3.5 Tumor characteristics

Among primary tumor measures (**Figure 2**), tumor greatest dimension did not show a clear prognostic association (one study; aHR = 1.02, 95% CI 0.27–3.80). Tumor size per centimeter was significantly associated with worse outcomes (three studies; aHR = 1.01, 95% CI 1.00–1.03; I² = 96.13%). Tumor thickness was examined in one study (aHR = 1.50, 95% CI 0.19–11.61), which does not allow firm conclusions. Depth of invasion was significantly associated with poorer survival (two studies; aHR = 1.03, 95% CI 1.01–1.05; I² = 0%).

### 3.6 Pathologic characteristics

Histologic grade (**Figure 2**) showed a non-significant association in the single study reporting it (aHR = 4.19, 95% CI 0.97–18.07). High-grade tumors (grade 3–4) demonstrated a significantly increased risk (two studies; aHR = 2.15, 95% CI 1.52–2.77; I² = 0%). Moderately differentiated tumors were assessed in one study (aHR = 2.08, 95% CI 0.79–5.52), and moderate-to-poor differentiation likewise relied on a single, non-conclusive estimate (aHR = 2.80, 95% CI 0.75–10.42). Poorly differentiated tumors, evaluated through one study, showed a strong significant association with worse outcomes (aHR = 7.75, 95% CI 2.61–23.01).

Patterns of invasion were examined in one study only (aHR = 2.115, 95% CI 0.79–5.61), providing insufficient evidence for definitive interpretation. Lymphovascular invasion was borderline and non-significant (two studies; aHR = 2.15, 95% CI –0.12 to 4.42; I² = 0%). Perineural invasion (three studies) did not reach statistical significance (aHR = 1.52, 95% CI –1.46 to 4.51; I² = 0%). Extension beyond the organ of origin was evaluated in one study (aHR = 1.86, 95% CI 1.00–3.44), suggesting a possible association but requiring replication. Positive surgical margins were assessed across three studies and showed no statistically significant association (aHR = 1.58, 95% CI 0.99–2.16; I² = 0%).

### 3.7 TNM status

**Figure 3:**
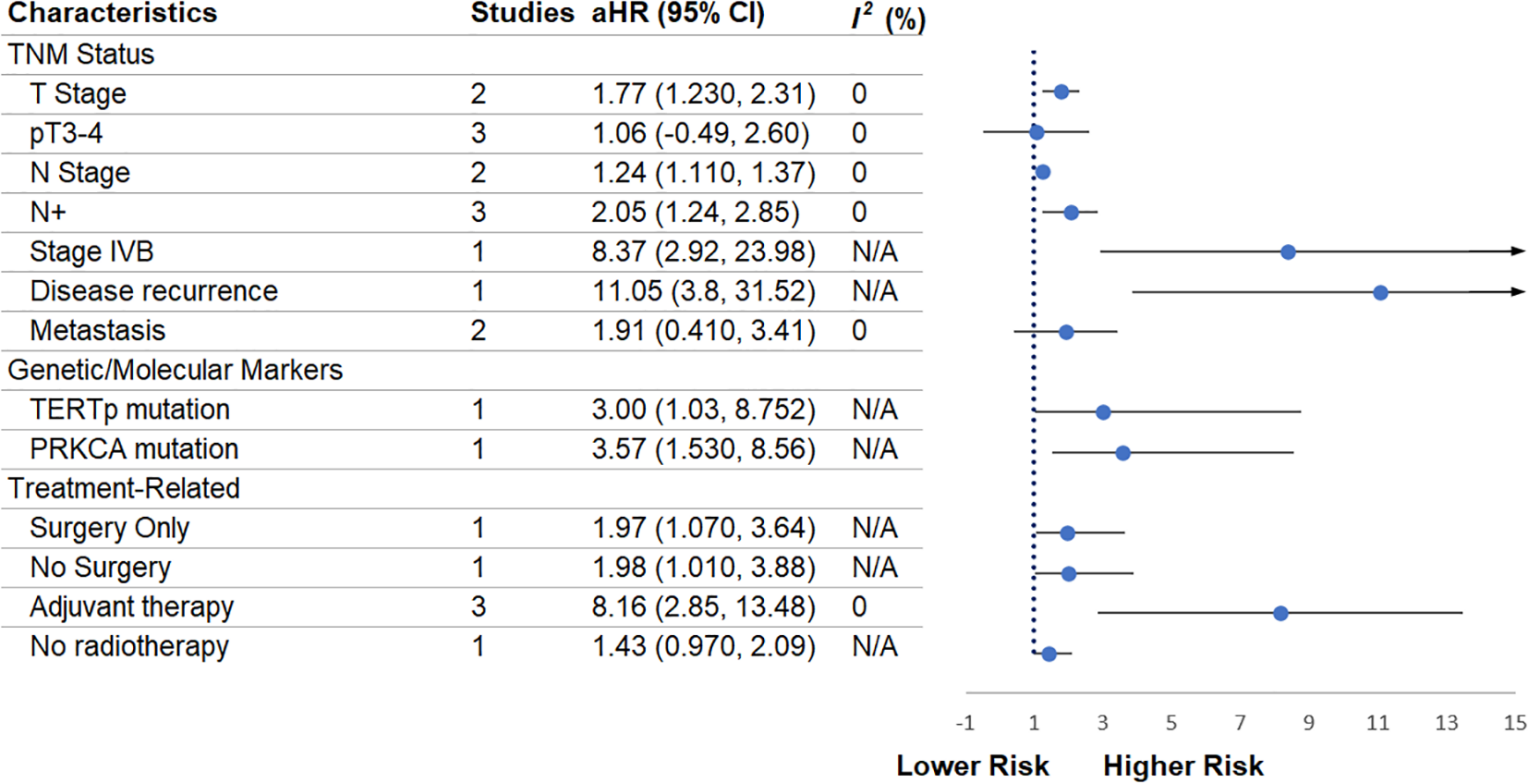
Forest plot showing the meta-analytic estimates of tumor stage, genetic markers, and treatment-related prognosticators of overall survival.

Advancing T stage (**Figure 3**) was significantly associated with poorer survival (two studies; aHR = 1.77, 95% CI 1.23–2.31; I² = 0%). Similarly, pT3–4 tumors demonstrated an elevated but non-significant risk (three studies; aHR = 1.06, 95% CI -0.49 – 2.60; I² = 0%). Higher N stage was significantly associated with worse outcomes (two studies; aHR = 1.24, 95% CI 1.11–1.37; I² = 0%), and the presence of nodal metastasis (N+) conferred a markedly increased risk (three studies; aHR = 2.05, 95% CI 1.24–2.85; I² = 0%).

Stage IVB was assessed in one study only and showed a substantially elevated hazard (aHR = 8.37, 95% CI 2.92–23.98), although this finding remains non-conclusive due to reliance on a single dataset. Disease recurrence, likewise reported in one study, demonstrated a strong association with worse prognosis (aHR = 11.05, 95% CI 3.8–31.52). Distant metastasis was analyzed across two studies and was not significantly associated with survival (aHR = 1.91, 95% CI 0.41–3.41; I² = 0%).

### 3.8 Genetic and molecular markers

TERTp mutation status (**Figure 3**) was evaluated in one study and showed a significant association with poorer outcomes (aHR = 3.00, 95% CI 1.03–8.752); however, conclusions remain limited given the single-study evidence base. PRKCA mutation also demonstrated a significant association (one study; aHR = 3.57, 95% CI 1.53–8.56), but similarly should be interpreted cautiously due to non-replication across studies.

### 3.9 Treatment-related factors

The choice of primary treatment modality showed notable associations with outcomes (**Figure 3**). Surgery alone was associated with increased risk (one study; aHR = 1.97, 95% CI 1.07–3.64), although this estimate is based on a single study. Conversely, omission of surgery showed a comparable magnitude of increased risk (one study; aHR = 1.98, 95% CI 1.01–3.88), also non-conclusive due to single-study origin. Receipt of adjuvant therapy was strongly associated with poorer survival across three studies (aHR = 8.16, 95% CI 2.85–13.48; I² = 0%), a finding likely reflecting treatment selection for patients with more advanced disease. Lack of radiotherapy was evaluated in one study and showed no significant association (aHR = 1.43, 95% CI 0.97–2.09).

## 4 Discussion

This systematic review and meta-analysis synthesized adjusted prognostic estimates from 24 studies on young-onset oral cancer and identified a pattern in which tumor biology and stage-related variables — rather than simple chronological age — appear to drive outcomes. Our principal findings were: 1) demographic variables such as younger age and female sex did not show consistent associations with overall survival after adjustment, while Black race was associated with worse prognosis; though not conclusive as this observation was based on a single study; 2) tumour burden and biologic aggressiveness (tumour size per cm, depth of invasion, higher T and N stage, nodal metastasis, and poor histologic grade) were the most robust and reproducible predictors of worse survival; 3) several molecular alterations (e.g. TERTp, PRKCA in single studies) and treatment-selection signals (receipt of adjuvant therapy) showed associations with poorer outcomes but were limited to single-study evidence and likely reflect confounding by indication. These results align with, and extend, the conclusions drawn in recent focused reviews and large pooled analyses of young-onset oral/tongue cancer (2, 3, 14, 15).

### 4.1 Interpretation in the context of existing evidence

Our finding that conventional tumour factors (size, depth of invasion, T/N stage, nodal positivity, high histologic grade) are associated with poorer survival in younger cohorts is concordant with multiple prior systematic reviews and population studies that emphasize stage and pathological features as primary determinants of outcome across age groups (2, 3, 41). Where prior pooled work has yielded apparently conflicting conclusions about whether younger patients do better or worse overall, those differences are often explained by differences in outcome definition, the inclusion of oropharyngeal subsites, and whether analyses used adjusted estimates. For example, Tagliabue et al. (3) specifically demonstrated that unadjusted comparisons frequently mask the adverse prognostic weight of comorbidity and stage, and that after adjustment older age was associated with worse mortality in tongue cancer, while younger patients had higher local recurrence risk. Similarly, Panda et al. (15) observed better crude overall survival for younger patients but worse disease-free survival and higher recurrence and distant metastasis rates in unmatched analyses, highlighting the importance of confounder control and subsite composition.

The consistent prognostic effect of nodal disease and higher T stage in our pooled analyses mirrors well-established biologic reality and staging data (e.g., AJCC/TNM) and is reinforced by contemporary prognostic-marker reviews for tongue cancer that show depth of invasion and nodal status to be among the strongest clinicopathologic predictors of outcome. Importantly, our pooled effect sizes for nodal metastasis (aHR ≈2.05) and advancing T stage (aHR ≈1.77) are clinically meaningful and concordant with those primary studies and meta-analyses that focus on oral tongue tumors (3, 16).

Molecular and biomarker data in young cohorts remain preliminary. Single-study associations identified in our review (e.g., TERTp and PRKCA mutations) indicate potential biological differences in some tumors arising at younger ages; however, these findings are not yet replicated across independent cohorts and therefore cannot be considered established prognostic markers. This cautious conclusion aligns with recent narrative syntheses and biomarker-focused meta-analyses that call for multicenter validation before adopting such markers clinically (14, 16).

### 4.2 Strengths of this review

This study pooled adjusted hazard ratios wherever available and prioritized regression-based, confounder-controlled estimates; that approach reduces bias from simple crude comparisons and from treatment selection effects. By focusing on adjusted estimates and using REML random-effects pooling, the synthesis emphasizes effect sizes that are more likely to reflect independent prognostic contributions. Our findings are therefore complementary to prior syntheses that pooled mainly unadjusted or mixed estimates (2, 15).

### 4.3 Limitations and risk of bias in the evidence base

Despite methodological strengths, the available evidence has important limitations that must temper interpretation. Although the present review focused on OSCC, which represents the dominant histology in young-onset oral cancer, heterogeneity in molecular drivers within OSCC could not be systematically explored due to limited reporting. Most included studies were retrospective, observational, and hospital-based; only a minority reported comprehensive confounder adjustment, and residual confounding (for example by comorbidity, socioeconomic status, access to care, or detailed treatment factors) remains likely. Several prognostic associations were based on single-study estimates (e.g., specific mutations, some histologic subcategories), precluding confident generalization. The NIH quality assessment applied across studies highlighted frequent deficiencies in controlling for confounders and in analytic transparency; these concerns mirror the assessments reported in prior reviews.

Heterogeneity across studies was another important limitation. Studies used different age cutoffs to define “young,” varied in subsite inclusion (oral cavity vs. oropharynx; tongue-only analyses versus mixed oral sites), and differed in stage distribution and treatment approaches, all of which contribute to statistical heterogeneity and limit pooled inference. Several earlier systematic reviews have underlined the same source of heterogeneity and recommendation for standardized definitions (3, 14, 15).

In addition, although several studies reported adjusted associations with disease-specific or disease-free survival, the limited number of studies per outcome and inconsistent reporting precluded quantitative synthesis; these findings are therefore presented narratively in **Supplementary Table S3**.

Finally, several potentially important prognostic domains (e.g., HPV status in subsites, comprehensive genomic signatures, immunologic markers, and detailed margin and nodal-yield metrics) were either inconsistently reported or absent in most studies; this limited our ability to perform subgroup or meta-regression analyses that might identify effect modification by these factors. Recent narrative syntheses and methodological reviews reach similar conclusions about evidence gaps in molecular and prognostic modelling for younger patients.

### 4.4 Clinical and research implications

For clinicians, the principal implication is that established tumour factors (size, depth, stage, nodal metastasis, poor differentiation) should remain central to risk stratification and treatment planning in younger patients rather than using chronological age alone as the justification for de-escalation or escalation of therapy. Our pooled results suggest that young patients with adverse pathologic features carry similar or higher risks as older counterparts and therefore warrant guideline-concordant staging, neck management, and adjuvant decision-making based on established pathologic indicators (2, 3).

From a research standpoint, several priorities emerge. First, prospective, multi-institutional cohorts with pre-specified age thresholds and harmonized reporting of clinicopathologic, treatment, and molecular variables are needed to validate single-study molecular signals (e.g., TERTp, PRKCA) and to allow robust multivariable modelling. Second, prognostic model development for young-onset oral cancer should follow best-practice guidance (transparent reporting, adequate events per variable, internal/external validation) because current prognostic models for head and neck subsites frequently suffer high risk of bias and limited external validation. Finally, studies that separate oral cavity subsites (tongue vs. non-tongue) and that stratify by HPV status where relevant will reduce heterogeneity and produce clinically actionable stratification tools (14).

## 5 Conclusion

In this prognostic synthesis of young-onset oral cancer, established pathological markers of tumour burden and aggressiveness—rather than chronological youth—emerged as the most consistent predictors of adverse survival. Although some molecular findings are intriguing, they are not yet sufficiently replicated to inform practice. Future prospective, multicenter studies with harmonized definitions and comprehensive confounder control are required to validate putative molecular prognosticators and to develop trustworthy age-specific prognostic models. These steps will be necessary before age can be used as an independent criterion to alter standard-of-care treatment pathways.

## Data Availability

The original contributions presented in the study are included in the article/**Supplementary Material**. Further inquiries can be directed to the corresponding author.

## References

[1] Kumar V . A comprehensive review of risk factors, diagnosis, and treatment options for oral squamous cell carcinoma. Curr Cancer Ther Rev. (2025). doi: 10.2174/0115733947379301250703053245

[2] Kaminagakura E Tango RN Cruz-Perez D Bonan R Yamamoto de Almeida L de Almeida Lança ML . Oral squamous cell carcinoma outcome in adolescent/young adult: systematic review and M eta-analysis. Head Neck. (2022) 44:548–61. doi: 10.1002/hed.26940, PMID: 34808012

[3] Tagliabue M Belloni P De Berardinis R Gandini S Chu F Zorzi S . A systematic review and meta-analysis of the prognostic role of age in oral tongue cancer. Cancer Med. (2021) 10:2566–78. doi: 10.1002/cam4.3795, PMID: 33760398 PMC8026930

[4] Llewellyn CD Linklater K Bell J Johnson NW Warnakulasuriya S . An analysis of risk factors for oral cancer in young people: A case-control study. Oral Oncol. (2004) 40:304–13. doi: 10.1016/j.oraloncology.2003.08.015, PMID: 14747062

[5] Llewellyn C Johnson N Warnakulasuriya K . Risk factors for squamous cell carcinoma of the oral cavity in young people—a comprehensive literature review. Oral Oncol. (2001) 37:401–18. doi: 10.1016/S1368-8375(00)00135-4, PMID: 11377229

[6] Davidson BJ Root WA Trock BJ . Age and survival from squamous cell carcinoma of the oral tongue. Head Neck. (2001) 23:273–9. doi: 10.1002/hed.1030, PMID: 11400227

[7] Garavello W Spreafico R Gaini RM . Oral tongue cancer in young patients: A matched analysis. Oral Oncol. (2007) 43:894–7. doi: 10.1016/j.oraloncology.2006.10.013, PMID: 17307026

[8] Hilly O Shkedy Y Hod R Soudry E Mizrachi A Hamzany Y . Carcinoma of the oral tongue in patients younger than 30 years: comparison with patients older than 60 years. Oral Oncol. (2013) 49:987–90. doi: 10.1016/j.oraloncology.2013.07.005, PMID: 23927849

[9] Kourelis K Tsue T Girod D Tawfik O Sykes K Shnayder Y . Negative prognostic factors for head and neck cancer in the young. J BUON. (2013) 18:459–64., PMID: 23818362

[10] Park JO Sun DI Cho KJ Joo YH Yoo HJ Kim MS . Clinical outcome of squamous cell carcinoma of the tongue in young patients: A stage-matched comparative analysis. Clin Exp otorhinolaryngol. (2010) 3:161–5. doi: 10.3342/ceo.2010.3.3.161, PMID: 20978546 PMC2958509

[11] Zhang YY Wang DC Su JZ Jia LF Peng X Yu GY . Clinicopathological characteristics and outcomes of squamous cell carcinoma of the tongue in different age groups. Head Neck. (2017) 39:2276–82. doi: 10.1002/hed.24898, PMID: 28842932

[12] Bello IO Almangush A Heikkinen I Haglund C Coletta RD Kowalski LP . Histological characteristics of early-stage oral tongue cancer in young versus older patients: A multicenter matched-pair analysis. Oral Dis. (2020) 26:1081–5. doi: 10.1111/odi.13288, PMID: 31994277

[13] Sharma S Satyanarayana L Asthana S Shivalingesh K Goutham BS Ramachandra S . Oral cancer statistics in India on the basis of first report of 29 population-based cancer registries. J Oral Maxillofac Pathol. (2018) 22:18–26. doi: 10.4103/jomfp.JOMFP_113_17, PMID: 29731552 PMC5917535

[14] Lenoci D Moresco E Cavalieri S Bergamini C Torchia E Botta L . Oral cancer in young adults: incidence, risk factors, prognosis, and molecular biomarkers. Front Oncol. (2024) 14:1452909. doi: 10.3389/fonc.2024.1452909, PMID: 39421447 PMC11484398

[15] Panda S Mohanty N Panda S Mishra L Gopinath D Sahoo A . Are survival outcomes different for young and old patients with oral and oropharyngeal squamous cell carcinoma? A systematic review and meta-analysis. Cancers. (2022) 14:1886. doi: 10.3390/cancers14081886, PMID: 35454794 PMC9029651

[16] Almangush A Heikkinen I Mäkitie AA Coletta RD Läärä E Leivo I . Prognostic biomarkers for oral tongue squamous cell carcinoma: A systematic review and meta-analysis. Br J Cancer. (2017) 117:856–66. doi: 10.1038/bjc.2017.244, PMID: 28751758 PMC5589992

[17] Page MJ McKenzie JE Bossuyt PM Boutron I Hoffmann TC Mulrow CD . The prisma 2020 statement: an updated guideline for reporting systematic reviews. bmj. (2021) 372. doi: 10.1136/bmj.n71, PMID: 33782057 PMC8005924

[18] Adduri R Sr. Kotapalli V Gupta NA Gowrishankar S Srinivasulu M Ali MM . P53 nuclear stabilization is associated with fhit loss and younger age of onset in squamous cell carcinoma of oral tongue. BMC Clin Pathol. (2014) 14:37. doi: 10.1186/1472-6890-14-37, PMID: 25152695 PMC4141988

[19] Bommakanti KK Abiri A Han AY Goshtasbi K Kuan EC St John MA . Stage-specific survival in young patients with oral tongue squamous cell carcinoma. Otolaryngology--head Neck Surg. (2023) 168:398–406. doi: 10.1177/01945998221101191, PMID: 35700039

[20] Decker JM Filho OV Freitas MO Silva-Fernandes IJ Dantas TS Campêlo CS . Pms2: A potential prognostic protein marker in oral squamous cell carcinoma. Med oral patologia Oral y cirugia bucal. (2021) 26:e451–e8. doi: 10.4317/medoral.24303, PMID: 33247565 PMC8254887

[21] Deneuve S Guerlain J Dupret-Bories A Majoufre C Philouze P Ceruse P . Oral tongue squamous cell carcinomas in young patients according to their smoking status: A gettec study. Eur Arch oto-rhino-laryngol. (2022) 279:415–24. doi: 10.1007/s00405-021-06793-7, PMID: 33877432

[22] Fan Y Zheng L Mao MH Huang MW Liu SM Zhang J . Survival analysis of oral squamous cell carcinoma in a subgroup of young patients. Asian Pacific J Cancer prevention: APJCP. (2014) 15:8887–91. doi: 10.7314/apjcp.2014.15.20.8887, PMID: 25374224

[23] Farhat MC Dyalram D Ord RA Lubek JE . Oral squamous cell carcinoma in patients aged 45 and younger: prognosis, survival, and quality of life. Oral surgery Oral medicine Oral Pathol Oral Radiol. (2022) 133:518–25. doi: 10.1016/j.oooo.2021.08.023, PMID: 34758935

[24] Gamez ME Kraus R Hinni ML Moore EJ Ma DJ Ko SJ . Treatment outcomes of squamous cell carcinoma of the oral cavity in young adults. Oral Oncol. (2018) 87:43–8. doi: 10.1016/j.oraloncology.2018.10.014, PMID: 30527242

[25] Iype EM Pandey M Mathew A Thomas G Nair MK . Squamous cell cancer of the buccal mucosa in young adults. Br J Oral Maxillofac Surg. (2004) 42:185–9. doi: 10.1016/j.bjoms.2004.02.008, PMID: 15121260

[26] Iype EM Pandey M Mathew A Thomas G Sebastian P Nair MK . Oral Cancer among Patients under the Age of 35 Years. J postgraduate Med. (2001) 47:171–6. 11832617

[27] Kies MS Boatright DH Li G Blumenschein G El-Naggar AK Brandon Gunn G . Phase ii trial of induction chemotherapy followed by surgery for squamous cell carcinoma of the oral tongue in young adults. Head Neck. (2012) 34:1255–62. doi: 10.1002/hed.21906, PMID: 22009800 PMC3893095

[28] Kim S Lee C Kim H Yoon SO . Genetic characteristics of advanced oral tongue squamous cell carcinoma in young patients. Oral Oncol. (2023) 144:106466. doi: 10.1016/j.oraloncology.2023.106466, PMID: 37393663

[29] Lee SU Moon SH Choi SW Cho KH Park JY Jung YS . Prognostic significance of smoking and alcohol history in young age oral cavity cancer. Oral Dis. (2020) 26:1440–8. doi: 10.1111/odi.13432, PMID: 32430951

[30] Liao PH Lee CC . The influence of marital status on survival for patients aged 65 years and younger with oral cavity cancer. Auris nasus larynx. (2018) 45:1227–32. doi: 10.1016/j.anl.2018.03.007, PMID: 29685504

[31] Manuel S Raghavan SK Pandey M Sebastian P . Survival in patients under 45 years with squamous cell carcinoma of the oral tongue. Int J Oral Maxillofac Surg. (2003) 32:167–73. doi: 10.1054/ijom.2002.0271, PMID: 12729777

[32] Mascitti M Tempesta A Togni L Capodiferro S Troiano G Rubini C . Histological features and survival in young patients with hpv-negative oral squamous cell carcinoma. Oral Dis. (2020) 26:1640–8. doi: 10.1111/odi.13479, PMID: 32531817

[33] Miller C Shay A Tajudeen B Sen N Fidler M Stenson K . Clinical features and outcomes in young adults with oral tongue cancer. Am J Otolaryngol. (2019) 40:93–6. doi: 10.1016/j.amjoto.2018.09.022, PMID: 30472130

[34] Mneimneh WS Xu B Ghossein C Alzumaili B Sethi S Ganly I . Clinicopathologic characteristics of young patients with oral squamous cell carcinoma. Head Neck Pathol. (2021) 15:1099–108. doi: 10.1007/s12105-021-01320-w, PMID: 33797696 PMC8633158

[35] Myers JN Elkins T Roberts D Byers RM . Squamous cell carcinoma of the tongue in young adults: increasing incidence and factors that predict treatment outcomes. Otolaryngology--head Neck Surg. (2000) 122:44–51. doi: 10.1016/s0194-5998(00)70142-2, PMID: 10629481

[36] Okuyama K Yanamoto S Michi Y Shibata E Tsuchiya M Yokokawa M . Multicenter retrospective analysis of clinicopathological features and prognosis of oral tongue squamous cell carcinoma in adolescent and young adult patients. Medicine. (2021) 100:e27560. doi: 10.1097/md.0000000000027560, PMID: 34731158 PMC8519201

[37] Parzefall T Schnoell J Monschein L Foki E Liu DT Frohne A . Prkca overexpression is frequent in young oral tongue squamous cell carcinoma patients and is associated with poor prognosis. Cancers (Basel). (2021) 13:2082. doi: 10.3390/cancers13092082, PMID: 33923093 PMC8123332

[38] Subramaniam N Balasubramanian D Murthy S Vidhyadharan S Thankappan K Iyer S . Oral cancer in the young with no tobacco exposure: A distinct epidemiological subset? J Head Neck Physicians Surgeons. (2018) 6:86–92. doi: 10.4103/2347-8128.208524

[39] Thomas L Moore EJ McGree ME Olsen KD Kasperbauer JL Erickson LA . Prognostic features, human papillomavirus status, and epidermal growth factor receptor expression in oral squamous cell carcinoma in young adults. Am J Otolaryngol. (2012) 33:650–6. doi: 10.1016/j.amjoto.2012.01.009, PMID: 22387125

[40] Warnakulasuriya S Mak V Möller H . Oral cancer survival in young people in south east England. Oral Oncol. (2007) 43:982–6. doi: 10.1016/j.oraloncology.2006.11.021, PMID: 17350878

[41] de Morais EF Mafra RP Gonzaga AKG de Souza DLB Pinto LP da Silveira ÉJD . Prognostic factors of oral squamous cell carcinoma in young patients: A systematic review. J Oral Maxillofac Surg. (2017) 75:1555–66. doi: 10.1016/j.joms.2016.12.017, PMID: 28061358

