# Supplementary material for: Prognostic factors in young patients with oral cavity cancer: a systematic review and meta-analysis of 24 studies
Source: Front Oncol. 2026 Feb 25;16:1780074. doi: 10.3389/fonc.2026.1780074 (PMC12975552; doi:10.3389/fonc.2026.1780074)
Supplement: Supplementary file 1 [file Table1.docx]

**Table S1.** The search query employed in the database search

| No. | Search query | Results |
| --- | --- | --- |
| PubMed | | |
| #1 | Young*[tiab] OR "40 years"[tiab] OR "35 years"[tiab] OR "45 years"[tiab] OR "39 years"[tiab] OR "under 45"[tiab] OR “below 45”[tiab] OR adolescent*[tiab] OR "below 40"[tiab] OR "under 40"[tiab] OR younger[tiab] OR “below 35”[tiab] OR “under 35”[tiab] OR “under 39”[tiab] OR “below 39”[tiab] OR “40-year*”[tiab] OR “35-year*”[tiab] OR “39-year*”[tiab] OR “45-year*”[tiab] OR “under 30”[tiab] OR “below 30”[tiab] | 1413895 |
| #2 | “oral cancer”[tiab] OR “oral carcinoma”[tiab] OR “oral neoplasm”[tiab] OR “oral cavity cancer”[tiab] OR “oral cavity carcinoma”[tiab] OR “oral cavity neoplasm”[tiab] OR “mouth cancer”[tiab] OR “mouth carcinoma”[tiab] OR “mouth neoplasm”[tiab] OR “gingival cancer”[tiab] OR “gingival carcinoma”[tiab] OR “gingival neoplasm”[tiab] OR “lip cancer”[tiab] OR “lip carcinoma”[tiab] OR “lip neoplasm”[tiab] OR “tongue cancer”[tiab] OR “tongue carcinoma”[tiab] OR “tongue neoplasm”[tiab] OR “buccal cancer”[tiab] OR “buccal carcinoma”[tiab] OR “palate cancer”[tiab] OR “palate carcinoma”[tiab] OR “palate neoplasm”[tiab] OR “oral cavity squamous”[tiab] OR “oral squamous”[tiab] OR "Mouth Neoplasms"[Mesh] | 94627 |
| #3 | Prognostic*[tiab] OR regression[tiab] OR Cox[tiab] OR hazard*[tiab] OR “odds ratio”[tiab] OR “relative risk”[tiab] OR multivariate[tiab] OR multivariable[tiab] OR univariate[tiab] OR univariable[tiab] OR “risk factor*”[tiab] OR predict*[tiab] OR coefficient[tiab] | 4886553 |
| #4 | “this review”[tiab] OR “narrative review”[tiab] OR “literature review”[tiab] OR “systematic review”[tiab] OR “meta-analysis”[tiab] OR “meta analysis”[tiab] OR “case report”[tiab] | 1943870 |
| #5 | #1 AND #2 AND #3 NOT #4 | 793 |
| Scopus | | |
| #1 | TITLE-ABS-KEY (Young*) OR TITLE-ABS-KEY ("40 years") OR TITLE-ABS-KEY ("35 years") OR TITLE-ABS-KEY ("45 years") OR TITLE-ABS-KEY ("39 years") OR TITLE-ABS-KEY ("under 45") OR TITLE-ABS-KEY ( “below 45”) OR TITLE-ABS-KEY (adolescent*) OR TITLE-ABS-KEY ("below 40") OR TITLE-ABS-KEY ("under 40") OR TITLE-ABS-KEY (younger) OR TITLE-ABS-KEY ( “below 35”) OR TITLE-ABS-KEY (“under 35”) OR TITLE-ABS-KEY (“under 39”) OR TITLE-ABS-KEY (“below 39”) OR TITLE-ABS-KEY (“40-year*”) OR TITLE-ABS-KEY (“35-year*”) OR TITLE-ABS-KEY (“39-year*”) OR TITLE-ABS-KEY (“45-year*”) OR TITLE-ABS-KEY (“under 30”) OR TITLE-ABS-KEY (“below 30”) | 4964346 |
| #2 | TITLE-ABS-KEY (“oral cancer”) OR TITLE-ABS-KEY (“oral carcinoma”) OR TITLE-ABS-KEY (“oral neoplasm”) OR TITLE-ABS-KEY (“oral cavity cancer”) OR TITLE-ABS-KEY (“oral cavity carcinoma”) OR TITLE-ABS-KEY (“oral cavity neoplasm”) OR TITLE-ABS-KEY (“mouth cancer”) OR TITLE-ABS-KEY (“mouth carcinoma”) OR TITLE-ABS-KEY (“mouth neoplasm”) OR TITLE-ABS-KEY (“gingival cancer”) OR TITLE-ABS-KEY (“gingival carcinoma”) OR TITLE-ABS-KEY (“gingival neoplasm”) OR TITLE-ABS-KEY (“lip cancer”) OR TITLE-ABS-KEY (“lip carcinoma”) OR TITLE-ABS-KEY (“lip neoplasm”) OR TITLE-ABS-KEY (“tongue cancer”) OR TITLE-ABS-KEY (“tongue carcinoma”) OR TITLE-ABS-KEY (“tongue neoplasm”) OR TITLE-ABS-KEY (“buccal cancer”) OR TITLE-ABS-KEY (“buccal carcinoma”) OR TITLE-ABS-KEY (“palate cancer”) OR TITLE-ABS-KEY (“palate carcinoma”) OR TITLE-ABS-KEY (“palate neoplasm”) OR TITLE-ABS-KEY (“oral cavity squamous”) OR TITLE-ABS-KEY (“oral squamous”) | 90726 |
| #3 | TITLE-ABS-KEY (Prognostic*) OR TITLE-ABS-KEY (regression) OR TITLE-ABS-KEY (Cox) OR TITLE-ABS-KEY (hazard*) OR TITLE-ABS-KEY (“odds ratio”) OR TITLE-ABS-KEY (“relative risk”) OR TITLE-ABS-KEY (multivariate) OR TITLE-ABS-KEY (multivariable) OR TITLE-ABS-KEY (univariate) OR TITLE-ABS-KEY (univariable) OR TITLE-ABS-KEY (“risk factor*”) OR TITLE-ABS-KEY (predict*) OR TITLE-ABS-KEY (coefficient) | 12758980 |
| #4 | TITLE-ABS-KEY (“this review”) OR TITLE-ABS-KEY (“narrative review”) OR TITLE-ABS-KEY (“literature review”) OR TITLE-ABS-KEY (“systematic review”) OR TITLE-ABS-KEY (“meta-analysis”) OR TITLE-ABS-KEY (“meta analysis”) OR TITLE-ABS-KEY (“case report”) | 5302467 |
| #5 | #1 AND #2 AND #3 AND NOT #4 | 3007 |
|  | Filters: human, English, Medicine, Article | 2053 |
| Web of Science | | |
| #1 | AB=Young* OR AB="40 years" OR AB="35 years" OR AB="45 years" OR AB="39 years" OR AB="under 45" OR AB= “below 45” OR AB=adolescent* OR AB="below 40" OR AB="under 40" OR AB=younger OR AB= “below 35” OR AB=“under 35” OR AB=“under 39” OR AB=“below 39” OR AB=“40-year*” OR AB=“35-year*” OR AB=“39-year*” OR AB=“45-year*” OR AB=“under 30” OR AB=“below 30” | 1682678 |
| #2 | AB=“oral cancer” OR AB=“oral carcinoma” OR AB=“oral neoplasm” OR AB=“oral cavity cancer” OR AB=“oral cavity carcinoma” OR AB=“oral cavity neoplasm” OR AB=“mouth cancer” OR AB=“mouth carcinoma” OR AB=“mouth neoplasm” OR AB=“gingival cancer” OR AB=“gingival carcinoma” OR AB=“gingival neoplasm” OR AB=“lip cancer” OR AB=“lip carcinoma” OR AB=“lip neoplasm” OR AB=“tongue cancer” OR AB=“tongue carcinoma” OR AB=“tongue neoplasm” OR AB=“buccal cancer” OR AB=“buccal carcinoma” OR AB=“palate cancer” OR AB=“palate carcinoma” OR AB=“palate neoplasm” OR AB=“oral cavity squamous” OR AB=“oral squamous” | 31312 |
| #3 | AB=Prognostic* OR AB=regression OR AB=Cox OR AB=hazard* OR AB=“odds ratio” OR AB=“relative risk” OR AB=multivariate OR AB=multivariable OR AB=univariate OR AB=univariable OR AB=“risk factor*” OR AB=predict* OR AB=coefficient | 8618986 |
| #4 | AB=“this review” OR AB=“narrative review” OR AB=“literature review” OR AB=“systematic review” OR AB=“meta-analysis” OR AB=“meta analysis” OR AB=“case report” | 1781699 |
| #5 | #1 AND #2 AND #3 NOT #4 | 461 |

**Table S2.** A summary of the (crude) prognostic factors in young oral cancer patients as reported by included studies

| Study (YOP) | Outcome Measure | Predictor | HR | 2.5% CI | 97.5% CI |
| --- | --- | --- | --- | --- | --- |
| Liao (2018) | DSS | Age | 1 | 0.98 | 1.02 |
|  |  | Gender (Female) | 1.35 | 0.73 | 2.5 |
|  |  | Marital status (Unmarried) | 1.89 | 1.34 | 2.68 |
|  |  | Margin (Positive) | 1.76 | 0.95 | 3.27 |
|  |  | Thickness (>5mm) | 0.97 | 0.68 | 1.37 |
|  |  | Perineural invasion | 1.07 | 0.71 | 1.63 |
|  |  | Lymphovascular invasion | 1.41 | 0.72 | 2.78 |
|  |  | T category (T3-4) | 2.96 | 2.11 | 4.15 |
|  |  | N category (N2-3) | 4.79 | 3.36 | 6.82 |
|  |  | Stage (III-IV) | 3.79 | 2.66 | 5.39 |
|  |  | Differentiation (Poorly/undifferentiated) | 2.61 | 1.62 | 4.2 |
|  |  | Neck dissection (Yes) | 2.15 | 1.24 | 3.75 |
|  |  | Adjuvant therapy (Chemotherapy/radiotherapy) | 2.24 | 1.59 | 3.14 |
| Parzefall (2021) | OS | Age | 1.01 | 0.99 | 1.03 |
|  |  | T class | 1.86 | 1.41 | 2.44 |
|  |  | N class | 1.38 | 1.15 | 1.64 |
|  |  | PRKCA | 3.62 | 1.18 | 11.45 |
|  |  | Smoking | 1.54 | 0.75 | 4.45 |
|  |  | Sex | 1.08 | 0.89 | 1.24 |
|  | DFS | Age | 0.98 | 0.95 | 1.01 |
|  |  | T class | 0.72 | 0.34 | 1.49 |
|  |  | N class | 0.58 | 0.23 | 1.39 |
|  |  | PRKCA | 2.82 | 0.78 | 10.1 |
|  |  | Smoking | 0.98 | 0.68 | 5.03 |
|  |  | Sex | 1.24 | 0.66 | 3.32 |

YOP: year of publication; DSS: disease-specific survival; OS: overall survival; DFS: disease-free survival; HR: hazards ratio; CI: confidence interval.

**Table S3.** A summary of the adjusted prognostic factors (not eligible for meta-analysis) in young oral cancer patients as reported by included studies

| Study (YOP) | Outcome Measure | Predictor | HR | 2.5% CI | 97.5% CI |
| --- | --- | --- | --- | --- | --- |
| Adduri (2014) | DSS | P53 nuclear stabilization (NS+) | 3.35.1 | 1.8293 | 6.135 |
|  |  | P53 mutation (No) | 0.4274 | 0.1811 | 1.0084 |
|  |  | FHIT LOH (No) | 0.4967 | 0.2265 | 1.0893 |
| Liao (2018) | DSS | Marital status (Unmarried) | 1.51 | 1.06 | 2.16 |
|  |  | N category (N2-3) | 3.42 | 2.35 | 4.99 |
|  |  | Neck dissection (Yes) | 1.26 | 0.7 | 2.28 |
| Mneimneh (2021) | DSS | Age | 0.136 | 0.033 | 0.56 |
|  |  | Histologic grade | 6.753 | 1.345 | 33.912 |
|  |  | AJCC T stage | 1.484 | 0.573 | 3.841 |
|  |  | AJCC N Stage | 1.404 | 0.753 | 2.615 |
|  |  | Tumor greatest dimension | 1.124 | 0.276 | 4.58 |
| Subramaniam (2018) | NRFS | Extrandoal extension (Yes) | 1.85 | 0.775 | 1.175 |
